# Alaska Candles

**Published:** 1874-11

**Authors:** 


Alaska Candles.—A remarkable fish is said to be found in great numbers in the coast rivers of Alaska. It is about eight inches long, transparent, and the fattest of all the finny tribe. This fat, however, has not the oily, rancid taste of other fish, but is like fresh lard. When these fish are dried, the Indians often turn them to a novel and practical account—burn them in place of candles. They give a clear, brilliant light, and are not liable to be blown out by the wind. Mr. Manson, Superintendent at Fort Simpson, says that the tail should be lighted instead of the head, and each fish will burn about fifteen minutes.—*The Druggists' Circular.*

